# Transforming Diagnostics: A Comprehensive Review of Advances in Digital Pathology

**DOI:** 10.7759/cureus.71890

**Published:** 2024-10-19

**Authors:** Ghizal Fatima, Hekmat Alhmadi, Abbas Ali Mahdi, Najah Hadi, Jan Fedacko, Aminat Magomedova, Sidrah Parvez, Ammar Mehdi Raza

**Affiliations:** 1 Biotechnology, Eras Lucknow Medical College and Hospital, Lucknow, IND; 2 Chemistry, Al-Muthanna University, Samawah, IRQ; 3 Biotechnology, Era University, Lucknow, IND; 4 Medicine, Kufa University, Najaf, IRQ; 5 Cardiology, Pavol Jozef Šafárik University, Kosice, SVK; 6 Biostatistics, Lomonosov Moscow State University, Moscow, RUS; 7 Pediatric Dentistry, Career Dental College and Hospital, Lucknow, IND

**Keywords:** artificial intelligence, diagnostic accuracy, digital pathology, personalized medicine, telepathology, whole slide imaging

## Abstract

Digital pathology has emerged as a revolutionary field, transforming traditional diagnostic practices by integrating advanced imaging technologies, computational tools, and artificial intelligence (AI). Adopting digital slides over conventional glass slides enables high-resolution imaging, facilitating remote consultations, second opinions, and telepathology. The digitalization of pathology laboratories enhances workflow efficiency and allows for large-scale data storage, retrieval, and analysis, paving the way for developing robust diagnostic algorithms. One of the most transformative aspects of digital pathology is its synergy with AI and machine learning (ML). These technologies have enabled the automation of repetitive processes, including diseased feature detection, biomarker quantification, and tissue segmentation. This has decreased inter-observer variability and increased diagnostic accuracy. AI-driven algorithms are particularly beneficial in complex cases, assisting pathologists in detecting subtle patterns that might be missed through manual examination.

Furthermore, digital pathology plays a critical role in personalized medicine by enabling the precise characterization of tumors, which leads to targeted therapy decisions. Integrating digital pathology with genomics and other omics data holds promise for a more holistic understanding of diseases, driving innovation in diagnostics and treatment. However, the transition to digital pathology is challenging. Issues such as data standardization, regulatory compliance, and the need for robust IT infrastructure must be addressed to realize its full potential. This review provides a detailed examination of these advances, their clinical applications, and the challenges faced in the widespread adoption of digital pathology. As the field continues to evolve, it is poised to play a pivotal role in shaping the future of diagnostics, offering new possibilities for improving patient outcomes. This comprehensive review explores the significant advances in digital pathology, highlighting its impact on diagnostics, research, and patient care.

## Introduction and background

The field of digital pathology is at the forefront of a significant transformation in the field of diagnostic medicine. Traditionally, pathology has relied on examining glass slides under a microscope, a practice that has been fundamental to diagnosing diseases for over a century. However, the advent of digital technologies has brought about a paradigm shift, enabling the conversion of glass slides into high-resolution digital images that can be stored, analyzed, and shared electronically [1]. This evolution from analog to digital has profound implications for how pathology is practiced and has opened up new avenues for enhancing diagnostic accuracy, efficiency, and collaboration. Various technological advancements support the shift to digital pathology. Developing whole slide imaging (WSI) systems, which allow for the digitization of glass slides at high resolution, is a key innovation. These digital slides can be easily archived, retrieved, and analyzed using sophisticated image analysis software. The ability to zoom in and out of these digital images and annotate specific areas enhances the precision and depth of analysis, providing pathologists with powerful tools to support their diagnostic decisions [2].

One of the most significant impacts of digital pathology is its potential to improve diagnostic accuracy. Traditional microscopy is subject to inter-observer variability, where different pathologists might interpret the same slide differently. Digital pathology solves this problem by allowing the utilization of computer-aided diagnostic (CAD) tools, which use artificial intelligence (AI) and machine learning (ML) algorithms to help pathologists identify and quantify pathological features. These algorithms can be trained to recognize patterns in tissue samples, such as the presence of tumor cells, which may not be easily discernible through manual examination. By augmenting the pathologist's expertise with AI, digital pathology has the potential to reduce diagnostic errors and ensure more consistent and accurate results [3].

Moreover, digital pathology facilitates remote consultations and telepathology, breaking down geographical barriers and allowing for expert opinions to be sought anywhere in the world. This capability is particularly valuable in regions with limited access to specialized pathologists. Telepathology enables real-time consultations, second opinions, and collaborative case reviews, enhancing patients' quality of care regardless of location. The ability to share digital slides easily also supports educational initiatives, enabling the dissemination of knowledge and the training of future pathologists in a more interactive and accessible manner [4]. In addition to enhancing diagnostic accuracy and collaboration, digital pathology plays an increasingly important role in research. The digitization of slides enables large-scale data collection and analysis, which is essential for developing new diagnostic markers and understanding disease mechanisms. Integrating digital pathology with other omics technologies, such as genomics and proteomics, leads to new insights into the molecular underpinnings of diseases, paving the way for personalized medicine. By correlating pathological findings with genetic data, researchers can identify specific biomarkers that can guide treatment decisions and improve patient outcomes [5].

**Figure 1:**
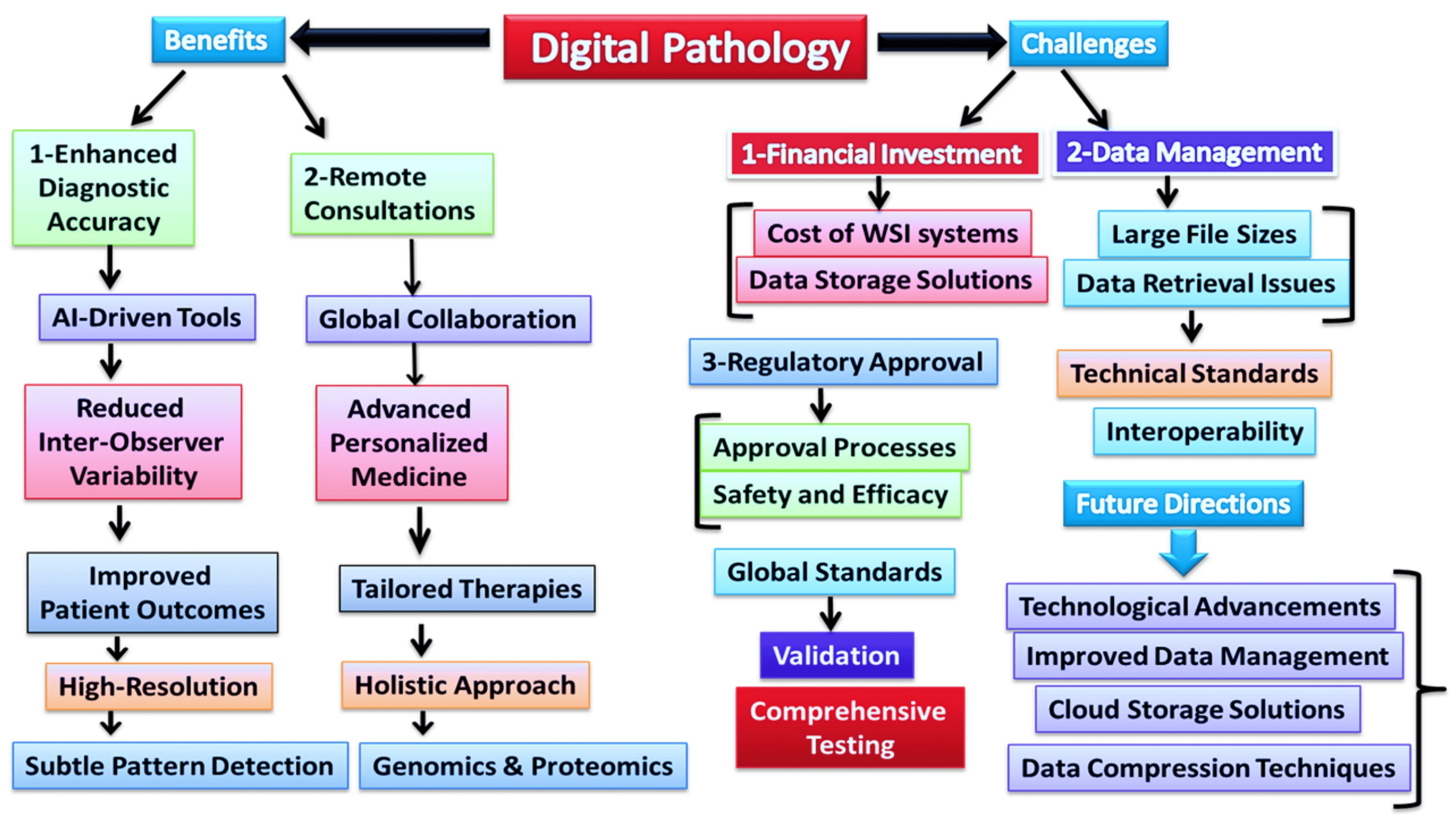
Digital pathology in revolutionizing diagnostics Providing high-resolution digital images improves diagnostic precision through AI-driven tools that reduce inter-observer variability and detect subtle patterns. This technology also facilitates remote consultations and global collaboration, bridging gaps in regions with limited access to specialized expertise. However, challenges such as significant financial investment, large data file management, and regulatory approval hurdles must be addressed. Financial constraints involve the costs of whole slide imaging systems and data storage solutions, while data management issues include handling large file sizes and ensuring efficient retrieval. Regulatory approval processes and the need for standardized image formats further complicate adoption. Future advancements in technology, improved data management, and establishing global standards are crucial for overcoming these challenges and fully realizing the potential of digital pathology in transforming healthcare. Image Credits: Dr. Ghizal Fatima

However, the widespread adoption of digital pathology is not without challenges. The transition requires significant investment in IT infrastructure, including the implementation of secure data storage systems and high-speed networks to handle the large file sizes associated with digital slides [6]. Standardizing image formats and data integration protocols is also critical to ensure interoperability between different systems and institutions. Additionally, there are regulatory considerations, as the use of digital pathology for primary diagnosis is still subject to approval by health authorities in many countries [7]. Digital pathology represents a transformative shift in the field of diagnostic medicine. By enhancing diagnostic accuracy, enabling remote consultations, and supporting research initiatives, it promises to improve patient care on a global scale [8]. However, realizing the full potential of digital pathology will require addressing the technical, regulatory, and logistic challenges accompanying its implementation. As these hurdles are overcome, digital pathology is set to play a pivotal role in the future of healthcare, offering new possibilities for innovation in diagnostics and treatment. Digital pathology is rapidly transforming the landscape of diagnostic medicine by integrating cutting-edge technologies with traditional practices. This review explores the field's key advancements, applications, and challenges, highlighting digital pathology's pivotal role in modern healthcare. Figure 1 illustrates digital pathology's impact, highlighting benefits like enhanced diagnostic accuracy and remote consultations. It also outlines challenges such as financial costs, data management, and regulatory approval. Future directions include technological advancements and improved data management, which are crucial for overcoming these challenges and advancing the field.

## Review

WSI technology

WSI represents a transformative technology at the heart of digital pathology, fundamentally altering how pathological specimens are examined, stored, and analyzed [9]. Traditionally, pathologists have relied on the microscopic examination of glass slides to diagnose diseases, a method that, while effective, comes with limitations in terms of accessibility, reproducibility, and the ability to share information across different geographical locations. WSI addresses these limitations by converting glass slides into high-resolution digital images, which can be stored, shared, and reviewed electronically, thereby enhancing the diagnostic process in numerous ways. The process of WSI involves scanning an entire tissue specimen on a glass slide to create a comprehensive digital image. This digital slide mimics the traditional glass slide but offers significant advantages. First, the image quality in WSI is often superior to what can be observed through a traditional microscope. The high-resolution nature of these digital images allows for a detailed examination of tissue architecture, cellular morphology, and other critical pathological features. Pathologists can zoom in and out of these images, much like they would with a microscope, but with the added benefit of digital tools that can enhance visualization and highlight specific areas of interest [10].

One of the primary advantages of WSI is its ability to facilitate remote consultations and collaborative diagnoses. In regions with limited access to specialized pathologists, WSI enables local healthcare providers to share digital slides with experts anywhere in the world, allowing for timely and accurate second opinions. This capability not only improves diagnostic accuracy but also speeds up the diagnostic process, which is critical in time-sensitive cases such as different forms of cancer. Additionally, the ease of sharing digital slides supports multidisciplinary team meetings, where specialists from different fields can review the same images simultaneously, leading to more informed and comprehensive patient care decisions. WSI is also essential for advancing research and the development of artificial intelligence (AI) in pathology. The ability to digitize slides creates vast datasets that can be used to train machine learning algorithms. These algorithms can be taught to recognize patterns, identify abnormalities, and even predict disease outcomes based on historical data. The large-scale WSI-generated data is crucial for developing and refining AI tools, which are increasingly integrated into the pathology workflow. These tools assist pathologists by automating routine tasks, such as tissue segmentation and cell counting, and provide diagnostic support through pattern recognition. The combination of WSI and AI has the potential to greatly enhance diagnostic accuracy and efficiency, reducing the workload on pathologists and enabling them to focus on more complex cases [11].

Moreover, WSI supports educational initiatives by allowing students and trainees to access high-quality digital slides from anywhere globally. This accessibility democratizes education, providing learners with the opportunity to study a wide variety of cases and gain experience with rare conditions that they might not encounter in their local practice. However, the adoption of WSI is not without challenges. The technology requires significant investment in scanning equipment, data storage solutions, and IT infrastructure to handle the large file sizes of digital slides. Additionally, there are ongoing concerns about data security and patient privacy, as well as the need for standardization in image formats and integration with existing laboratory information systems. WSI is a cornerstone of digital pathology, offering substantial benefits regarding image quality, remote collaboration, and the development of AI-driven diagnostic tools. As the technology continues to evolve and overcome current challenges, it is poised to play an increasingly vital role in both clinical practice and pathology research, ultimately improving patient outcomes and advancing the field of medicine [12].

Artificial intelligence and machine learning

Integrating AI and ML into digital pathology represents a transformative leap in diagnostic medicine, revolutionizing how pathologists interpret and analyze tissue samples. AI and ML technologies are increasingly being employed to automate routine tasks such as tissue segmentation, cell counting, and the identification of pathological features. These advancements not only enhance the efficiency of diagnostic workflows but also contribute significantly to the accuracy and consistency of diagnoses. One of the key benefits of AI-powered algorithms in pathology is their ability to reduce inter-observer variability, a common issue in traditional pathology. Variability in diagnoses can occur due to differences in individual pathologists' experience and interpretation, leading to inconsistencies in patient care. AI algorithms, trained on large datasets, provide standardized interpretations that help mitigate these discrepancies. For example, studies have demonstrated that AI can match or even surpass human pathologists in identifying certain cancer cells, such as those in breast and prostate cancers, thereby supporting more reliable diagnoses. AI also plays a crucial role in increasing diagnostic accuracy [13]. Machine learning models, particularly deep learning algorithms, have shown remarkable proficiency in identifying subtle patterns in tissue samples that human eyes might overlook. These patterns could include early signs of malignancy or other disease markers crucial for timely and accurate diagnosis of several diseases. By leveraging AI, pathologists can detect these early indicators, leading to more precise diagnoses and better patient outcomes. For instance, AI has been used to improve the detection of lymph node metastases in lung cancer, achieving higher sensitivity and specificity compared to traditional methods [14].

Moreover, AI and ML enable faster turnaround times for pathology reports. Traditional pathological analysis can be time-consuming, particularly in complex cases where multiple features must be examined. AI-driven tools can rapidly process and analyze large volumes of data, significantly reducing the time required to generate diagnostic reports. This is especially beneficial in busy clinical settings where timely diagnosis is critical for patient management [15].

However, the integration of AI into clinical practice is challenging. Deploying AI tools in pathology requires rigorous validation to ensure they are reliable and safe for clinical use. This includes extensive testing across diverse patient populations and tissue types to confirm that the AI algorithms can generalize well and produce accurate results consistently. Furthermore, regulatory approval is necessary before these tools can be widely adopted in clinical settings. Regulatory bodies such as the FDA have begun to establish guidelines for evaluating AI in medical devices, but the path to approval can be complex and time-consuming. Integrating AI and ML in digital pathology holds immense potential to enhance diagnostic accuracy, reduce variability, and improve efficiency in pathology workflows. As these technologies continue to evolve and undergo validation, they are poised to become indispensable tools in the practice of modern pathology, ultimately improving patient outcomes through more precise and timely diagnoses [16].

Telepathology and remote consultations

Telepathology, the practice of diagnosing diseases remotely through the use of digital images, has seen significant advancements with the advent of digital pathology. By leveraging high-resolution digital slides, telepathology enables pathologists to engage in real-time consultations, provide second opinions, and collaborate on complex cases without the constraints of physical location. This technological development has revolutionized the field of pathology, particularly benefiting regions with limited access to specialized medical expertise. One of the primary advantages of telepathology is its ability to bridge the gap between resource-rich and resource-limited areas. In many parts of the world, particularly in rural or underserved regions, access to specialized pathologists is limited or non-existent. Telepathology addresses this challenge by allowing digital slides to be shared instantaneously with experts located in different parts of the country or even the world. This capability ensures that patients in remote areas can receive high-quality diagnostic services, comparable to those available in major urban areas, without the need for expensive and time-consuming travel. The use of telepathology is particularly beneficial in scenarios requiring expert opinions, such as complex or rare cases where the local pathologist may seek a second opinion from a subspecialist [17]. Digital pathology platforms enable these consultations to occur rapidly, often in real-time, which is crucial in situations where prompt diagnosis is essential for effective treatment planning. The ability to quickly and efficiently consult with colleagues enhances the diagnostic process, leading to better patient outcomes in Neuropathologic Intraoperative Consultations [18].

Telepathology also plays a vital role in fostering global collaboration in both research and education. The ease of sharing digital slides across institutions facilitates the exchange of knowledge and expertise on an unprecedented scale. Researchers can collaborate on multicenter studies, pooling data and insights from different populations to achieve more robust and generalizable findings. This global exchange accelerates the pace of scientific discovery and the development of new diagnostic and therapeutic strategies. In education, telepathology offers an invaluable tool for training the next generation of pathologists. Medical students and trainees can access a diverse array of digital slides, including rare and complex cases that they might not encounter in their local practice. This exposure to a broader spectrum of pathology enhances their learning experience and prepares them for a wide range of diagnostic challenges. Telepathology also allows for interactive teaching sessions, where instructors and students can examine and discuss digital slides in real time, regardless of their physical locations [19].

Moreover, telepathology supports the standardization of diagnostic practices across different regions. By facilitating the sharing of digital slides and the exchange of diagnostic criteria, it helps harmonize pathology practices globally, reducing variability in diagnoses and improving the overall quality of care. This standardization is particularly important in the context of clinical trials and research studies, where consistent diagnostic criteria are essential for valid and reliable results. However, the widespread adoption of telepathology comes with certain challenges [20]. These include ensuring the security and privacy of patient data during digital transmission, the need for robust IT infrastructure, and the potential resistance from professionals accustomed to traditional microscopy. Addressing these challenges is crucial for the successful integration of telepathology into routine clinical practice. Telepathology, enhanced by digital pathology, is transforming the field of diagnostic medicine by enabling remote consultations, fostering global collaboration, and supporting educational initiatives. Its ability to connect pathologists across distances ensures that high-quality diagnostic services are accessible to patients everywhere, contributing to more equitable and effective healthcare delivery worldwide [5].

Impact on diagnostic accuracy

Telepathology, the practice of diagnosing diseases remotely through the use of digital images, has seen significant advancements with the advent of digital pathology. By leveraging high-resolution digital slides, telepathology enables pathologists to engage in real-time consultations, provide second opinions, and collaborate on complex cases without the constraints of physical location. This technological development has revolutionized the field of pathology, particularly benefiting regions with limited access to specialized medical expertise. One of the primary advantages of telepathology is its ability to bridge the gap between resource-rich and resource-limited areas. Access to specialized pathologists is limited or non-existent in many parts of the world, particularly in rural or underserved regions. Telepathology addresses this challenge by allowing digital slides to be shared instantaneously with experts located in different parts of the country or even the world. This capability ensures that patients in remote areas can receive high-quality diagnostic services comparable to those available in major urban areas without needing expensive and time-consuming travel. The use of telepathology is particularly beneficial in scenarios requiring expert opinions, such as complex or rare cases where the local pathologist may seek a second opinion from a subspecialist [17]. Digital pathology platforms enable these consultations to occur rapidly, often in real-time, which is crucial in situations where prompt diagnosis is essential for effective treatment planning. The ability to quickly and efficiently consult with colleagues enhances the diagnostic process, leading to better patient outcomes in Neuropathologic Intraoperative Consultations [18].

Telepathology also plays a vital role in fostering global collaboration in both research and education. The ease of sharing digital slides across institutions facilitates the exchange of knowledge and expertise on an unprecedented scale. Researchers can collaborate on multicenter studies, pooling data and insights from different populations to achieve more robust and generalizable findings. This global exchange accelerates the pace of scientific discovery and the development of new diagnostic and therapeutic strategies. In education, telepathology offers an invaluable tool for training the next generation of pathologists. Medical students and trainees can access diverse digital slides, including rare and complex cases they might not encounter in their local practice. This exposure to a broader spectrum of pathology enhances their learning experience and prepares them for a wide range of diagnostic challenges. Telepathology also allows for interactive teaching sessions, where instructors and students can examine and discuss digital slides in real time, regardless of their physical locations [19].

Moreover, telepathology supports the standardization of diagnostic practices across different regions. Sharing digital slides and exchanging diagnostic criteria helps harmonize pathology practices globally, reducing diagnosis variability and improving the overall quality of care. This standardization is particularly important in the context of clinical trials and research studies, where consistent diagnostic criteria are essential for valid and reliable results. However, the widespread adoption of telepathology comes with certain challenges [20]. These include ensuring the security and privacy of patient data during digital transmission, the need for robust IT infrastructure, and the potential resistance from professionals accustomed to traditional microscopy. Addressing these challenges is crucial for successfully integrating telepathology into routine clinical practice. Telepathology, enhanced by digital pathology, is transforming the field of diagnostic medicine by enabling remote consultations, fostering global collaboration, and supporting educational initiatives. Its ability to connect pathologists across distances ensures that high-quality diagnostic services are accessible to patients everywhere, contributing to more equitable and effective healthcare delivery worldwide [5].

Advances in personalized medicine

Digital pathology plays an increasingly pivotal role in advancing personalized medicine by enabling the precise characterization of tissue samples and facilitating a more individualized approach to patient care. Integrating digital pathology with omics technologies such as genomics, proteomics, and metabolomics provides a comprehensive understanding of disease mechanisms, which is crucial for developing targeted therapies. One of the primary ways digital pathology contributes to personalized medicine is by identifying and analyzing specific biomarkers. Digital pathology allows for the detailed examination of tissue samples, enabling pathologists to identify biomarkers indicative of particular disease states or responses to treatment. For instance, digital slides can be used to detect specific molecular markers in cancer tissues, such as hormone receptors or genetic mutations, which are critical for selecting appropriate targeted therapies. This capability is particularly valuable in oncology, where identifying biomarkers can guide the choice of targeted therapies and improve treatment outcomes for patients with cancers like breast, lung, and colorectal cancer [25].

Integrating digital pathology with other omic technologies enhances this process by providing a more holistic view of the disease. Genomics, which involves studying the complete set of DNA in an organism, can identify genetic mutations that contribute to disease development. When combined with digital pathology, genomics data can be mapped onto high-resolution digital images of tissue samples, allowing for the correlation of genetic alterations with specific histopathological features. Proteomics, the study of the proteome, is the entire set of proteins expressed by a genome, and it complements this by revealing how these genetic changes affect protein expression and function. This multi-layered approach helps understand how genetic and molecular alterations drive disease and how they can be targeted with specific treatments.

Moreover, digital pathology supports the development of precision medicine by enabling the stratification of patients based on their molecular profiles. This stratification allows for customized treatment plans according to each patient's disease's specific genetic and molecular characteristics. For example, patients with specific genetic mutations may benefit from targeted therapies designed to inhibit the activity of proteins produced by these mutations, while others may require different treatment approaches based on their unique molecular profiles [26].

Combining digital pathology with omics technologies also facilitates the development of new diagnostic and prognostic markers. By analyzing large datasets generated from digital slides and omics data, researchers can identify novel biomarkers that could be used for early detection, disease monitoring, and treatment response evaluation. This approach enhances the precision of diagnoses and contributes to developing personalized treatment regimens tailored to the individual patient. Digital pathology is crucial in advancing personalized medicine by enabling precise tissue characterization and integrating with omics technologies. This integration allows for a deeper understanding of disease mechanisms and supports the development of targeted therapies, ultimately leading to more individualized and effective patient care.

Advances and current limitations in cytological, histological, and IHC software implementations

Digital pathology has significantly enhanced cytological, histological, and immunohistochemical (IHC) analysis. However, the current software implementation level varies, with several promising developments yet to achieve widespread validation and clinical adoption. In cytology, integrating AI-based algorithms for automated cell detection and classification has shown promise in improving the sensitivity and specificity of diagnoses, particularly in screening programs for cervical and lung cancer [27]. However, fully automated cytological assessments are still in developmental phases, with challenges surrounding the heterogeneity of cytological samples and the need for high levels of manual oversight. Novel approaches using deep learning are currently being explored to refine the detection of subtle cellular abnormalities and reduce false positives, but more robust datasets and validation studies are required before these methods can be routinely applied in clinical settings.

In histology, digital pathology platforms have significantly improved image analysis through whole-slide imaging (WSI). This advancement allows pathologists to perform detailed tissue assessments with tools for measuring tumor margins, quantifying cell proliferation, and analyzing tissue architecture [28]. Despite these capabilities, the full potential of digital histopathology is hindered by the variability in tissue preparation and staining protocols, which can affect image quality and analysis outcomes. New approaches, such as integrating 3D histological reconstructions and multiplex staining, are being developed to address these limitations and provide deeper insights into tissue morphology.

In immunohistochemistry (IHC), digital pathology has enhanced the standardization of marker quantification, particularly for well-established markers like Ki-67 [29]. However, the current state of IHC software implementations is still evolving. While software-based analysis for certain markers has been validated, numerous other IHC markers lack the necessary validation for clinical use. Additionally, current algorithms often struggle with variations in staining intensity and background noise, leading to inconsistent results. Future innovations could focus on integrating AI to improve the accuracy of quantitative IHC analysis and develop universal protocols that standardize the staining and imaging process across laboratories. Looking forward, new approaches such as integrating multi-omics data (e.g., genomics, transcriptomics) with digital pathology platforms can potentially expand the diagnostic capabilities of software-driven analysis. However, these advances are still largely experimental and require significant validation before clinical application. Moreover, the digitization of pathology in resource-limited settings remains a distant goal, with the cost of infrastructure and the lack of trained personnel being major barriers.

Overall, while the technological revolution in digital pathology has introduced innovative methods in cytological, histological, and IHC analysis, the field continues to face challenges in validating, implementing, and standardizing these tools. Ongoing research and collaboration between software developers, pathologists, and regulatory bodies will be critical to advancing these methodologies and realizing the full potential of digital pathology in routine diagnostics.

Challenges and future directions

Despite its numerous benefits, the widespread adoption of digital pathology faces several challenges. The initial investment required for WSI systems, data storage, and IT infrastructure can be significant. Additionally, the large file sizes associated with digital slides require robust data management solutions to ensure efficient storage and retrieval. Standardizing image formats and data interoperability is also crucial for seamless integration across different systems and institutions. Regulatory approval is another hurdle, as health authorities in many countries still evaluate digital pathology use for primary diagnosis. Looking forward, the continued development of AI algorithms, improvements in data management, and the establishment of global standards will be key to overcoming these challenges. As digital pathology becomes more widely adopted, it has the potential to revolutionize the field of diagnostic medicine, offering new possibilities for improving patient care, advancing research, and reducing healthcare disparities [30].

Financial Investment

One of the primary barriers to widespread adoption is the substantial initial investment required for whole WSI systems, data storage solutions, and IT infrastructure. WSI systems, which convert glass slides into high-resolution digital images, are costly and require ongoing maintenance and upgrades. Additionally, the infrastructure needed to store and manage the large volumes of data generated by digital slides involves significant expenditures on servers, data storage solutions, and backup systems. Smaller laboratories and healthcare institutions may find these costs prohibitive, limiting the accessibility of digital pathology technologies [31].

Data Management and Interoperability

The large file sizes associated with digital slides necessitate robust data management solutions to ensure efficient storage, retrieval, and information sharing. Effective data management is critical to prevent data loss, ensure data integrity, and facilitate seamless access to digital slides across different platforms. Moreover, standardizing image formats and data interoperability is essential for integrating digital pathology systems across various institutions and systems. Without standardized protocols, sharing and comparing digital slides between different healthcare providers can be challenging, hindering collaboration and the potential benefits of digital pathology [32].

Regulatory Approval

Regulatory approval is another significant challenge, as health authorities in many countries still evaluate digital pathology's use for primary diagnosis. The process of gaining regulatory approval can be lengthy and complex, involving rigorous validation studies to ensure that digital pathology systems meet the required accuracy, reliability, and safety standards. Until clear guidelines and regulations are established, the adoption of digital pathology for diagnostic purposes may be limited [33].

Future Directions

Addressing these challenges requires ongoing efforts in several key areas. Continued development of AI algorithms is essential for improving diagnostic accuracy and reducing the workload on pathologists. Advances in AI can enhance the automation of routine tasks and support more accurate and efficient diagnoses. Improvements in data management technologies, such as cloud-based storage solutions and advanced data compression techniques, can help manage the large volumes of data generated by digital pathology systems. Establishing global standards for image formats, data interoperability, and regulatory requirements will facilitate the seamless integration of digital pathology across different systems and institutions. While digital pathology holds immense promise for revolutionizing diagnostic medicine, overcoming the challenges related to financial investment, data management, and regulatory approval is crucial for its widespread adoption. By addressing these challenges and continuing to advance technology, digital pathology has the potential to significantly improve patient care, advance research, and reduce healthcare disparities on a global scale.

## Conclusions

Digital pathology transforms diagnostics, particularly by enhancing accuracy through high-resolution imaging and AI integration. This technology improves diagnostic precision, reducing inter-observer variability, especially in complex cases like cancer. While advancements in remote consultations and collaborative reviews are bridging geographical gaps, the technology's potential in personalized medicine, such as integrating with other omics, remains in the early stages. For now, validation for immunohistochemical (IHC) markers is limited, with Ki-67 being one of the few in use. Additionally, molecular diagnostics cannot yet be fully realized through software alone. Challenges such as the high cost of whole-slide imaging (WSI) systems, regulatory approval for primary diagnosis, and lack of widespread adoption remain barriers, particularly in resource-limited regions. Despite these hurdles, continued technological improvements and global standardization efforts are essential for digital pathology to reach its full potential and play a key role in advancing healthcare.
